# Reinforcing Effects of the Synthetic Cathinone α-Pyrrolidinopropiophenone (α-PPP) in a Repeated Extended Access Binge Paradigm

**DOI:** 10.3389/fpsyt.2020.00862

**Published:** 2020-08-25

**Authors:** Erin K. Nagy, Paula F. Overby, M. Foster Olive

**Affiliations:** Addiction Neuroscience Laboratory, Department of Psychology, Arizona State University, Tempe, AZ, United States

**Keywords:** synthetic cathinone derivative, alpha-pyrrolidinopropiophenone, methylenedioxypyrovalerone, psychostimulant, self-administration, binge, extended access, rat

## Abstract

Synthetic cathinones are designer psychostimulants that are derivatives of the natural alkaloid cathinone, and produce effects similar to more traditional illicit stimulants such as cocaine and methamphetamine. The pyrovalerone cathinones methylenedioxypyrovalerone (MDPV) and α-pyrrolidinopropiophenone (α-PPP) exert their effects *via* inhibition of presynaptic dopamine and norepinephrine reuptake transporters. While the reinforcing effects of MDPV in rodents are well-established, very few studies have examined self-administration patterns of α-PPP. Users of synthetic cathinones often engage in repeated binge episodes of drug intake that last several days. We therefore sought to determine the reinforcing effects of three doses of α-PPP (0.05, 0.1 and 0.32 mg/kg/infusion) under conditions of prolonged binge-like access conditions, with three 96-h periods of drug access interspersed with 72 h of abstinence. MDPV (0.05 mg/kg/infusion) was used as a comparison drug. Our results show that both MDPV and the high (0.32 mg/kg/infusion) dose of α-PPP are readily self-administered at high levels across all three extended access periods, whereas lower doses of α-PPP produce variable and less robust levels of self-administration. These results indicate that higher doses of α-PPP have reinforcing effects under conditions of extended access, suggesting the potential for abuse and a need for consideration in drug control policies.

## Introduction

Frequently referred to as “bath salts”, synthetic cathinones are psychostimulants with neurochemical actions similar to those of cocaine and methamphetamine (1). As derivatives of the naturally occurring alkaloid cathinone isolated from *Catha edulis*, these drugs carry a high risk of adverse reactions such as agitated delirium, seizures, psychosis, organ failure, and death (2). Among the first cathinone derivatives to infiltrate drug markets in the 21^st^ century were the so-called “first generation” synthetic cathinones that included 3,4-methylenedioxypyrovalerone (MDPV), 4-methylmethcathinone (mephedrone), and methylone. However, due to the ease at which their chemical structures are modified, numerous other cathinone derivatives have emerged, including pyrovalerone cathinones (α-pyrrolidinophenones) such as α-pyrrolidinopentiophenone (α-PVP), α-pyrrolidinopropiophenone (α-PPP), amongst many others (3–5).

The pharmacological mechanisms of action of synthetic cathinones are similar in nature to those exerted by traditional psychostimulants (4, 6). For example, 4-methylmethcathinone (mephedrone) produces amphetamine-like monoamine releasing effects *via* inhibition and reversal of presynaptic transporters for norepinephrine and dopamine (NET and DAT, respectively) as well as the type 2 vesicular monoamine transporter (VMAT_2_). In contrast, MDPV, α-PVP and α-PPP produce cocaine-like inhibition of DAT and NET, with up to 100 times greater potency than cocaine and with longer durations of action (7, 8).

Studies in rodents support the notion that synthetic cathinones possess a high potential for abuse and dependence. For example, in line with their pharmacological actions, MDPV and α-PPP induce discriminative stimulus effects similar to those of cocaine and methylenedioxymethamphetamine (MDMA) (9–12) and are intravenously self-administered under conditions of limited drug access (13–15). However, detailed case reports have revealed that users of synthetic cathinones often engage in binge-like intake patterns that last 3-5 consecutive days or longer (16, 17). We recently characterized self-administration patterns of MDPV in rats using a prolonged binge-like intake paradigm, where several extended (96 h) drug access periods are separated by 72 h of abstinence in the home cage (18). This paradigm was originally developed to model multi-day binge-like intake of methamphetamine in rodents (19). The present study was designed to assess intake patterns of α-PPP under similar conditions of repeated periods of extended access to the drug.

## Materials and Methods

### Animals

Adult male Sprague-Dawley rats (300-350 g, Envigo, Placentia, CA) were used as subjects. Prior to surgical procedures, animals were housed in pairs in a vivarium on a reversed light-dark cycle (12:12; lights off at 0700 h), with temperature and humidity within guidelines of the National Institutes of Health. Following catheter implantation and recovery from surgery, rats were singly housed to prevent cagemate chewing and damage to vascular access ports. Food and water were available ad libitum at all times.

### Drugs

MDPV (Laboratory Supply USA, San Diego, CA) was verified to be >95% purity by liquid chromatography/mass spectrometry as we have previously reported (13). The same was true for initial experiments utilizing α-PPP, although additional amounts of this drug were purchased from Cayman Chemical (Ann Arbor, MI) in order to complete the study, as the original source was no longer available. Drugs were dissolved in 0.9% w/v sodium chloride for intravenous self-administration.

### Surgical Procedures

Rats were placed under anesthesia with isoflurane (5% induction, 2-3% maintenance) that was vaporized in O_2_ at a flow rate of 2 L/min. An incision was made on the neck to expose the jugular vein, and polyurethane catheters (Access Technologies, Skokie, IL) were inserted ~3.0 cm and secured with silk sutures. The other end of the catheter was routed subcutaneously to the dorsum, where it exited the skin *via* a 3-mm incision between the scapulae. This end of the catheter was connected to a vascular access port (Instech Laboratories, Plymouth Meeting, PA) that was secured to the surrounding skin with sutures. Following implantation, access ports were flushed with 0.2 ml of a Timentin solution (66.6 mg/ml, dissolved in saline containing 70 U/ml heparin). During postoperative care (5 days), rats received daily infusions of the heparinized Timentin solution to maintain catheter patency. During the first 3 days of operative care, rats were administered meloxicam (2 mg/kg s.c.) and buprenorphine (0.03 mg/kg s.c.) daily to reduce post-surgical discomfort. Animals then were randomly assigned to commence training for self-administration of either α-PPP, MDPV, or saline.

### Apparatus

Self-administration procedures were performed in operant conditioning chambers (Med Associates, Model ENV-007, St. Albans, VT) interfaced to a PC computer. Located at one end of each chamber was a 2.5-cm diameter active nosepoke aperture and a similarly sized inactive aperture. Within both apertures, a small LED provided visual cues during each drug infusion. Located at the top of the chamber was a speaker that provided a tone (~65 dB, 2900 Hz) during each drug delivery. Each conditioning chamber also contained a water bottle, and food pellets were placed on the floor of the chamber every morning of each 96-h session. Above each chamber was a liquid swivel that was connected to a PC-controlled syringe pump (Med Associates) for intravenous drug infusions. Drug solutions were delivered through polyethylene tubing housed within a metal tether that was connected to the vascular access port. Each operant chamber was located in a separate sound-attenuating cubicle that was equipped with a house light (programmed to match the light-dark cycle of the colony room), and a ventilation fan to mask external noise and odors.

### Self-Administration Procedures

Following surgical recovery, rats were allowed to spontaneously acquire self-administration of α-PPP, MDPV, or saline in 96-h sessions. Doses of 0.05, 0.1, and 0.32 mg/kg/infusion of α-PPP were selected based on a similar doses recently demonstrated to support self-administration in limited daily access sessions in rats (15). A dose of 0.05 mg/kg/infusion of MDPV was selected based on our prior findings of binge-like intake of this drug in the 96-h paradigm (18). After the first 96-h session, animals were removed from the operant chamber and returned to the home cage for 72 h of abstinence. This procedure of 96 h of drug access followed by 72 h of abstinence was repeated twice, so that each animal underwent a total of three 96-h self-administration sessions, each separated by 72 h of abstinence in the home cage. All reinforcers were available on a fixed-ratio 1 (FR1) schedule of reinforcement, where nosepokes into the active aperture lever resulted in reinforcer delivery in a volume of 0.06 ml over a 1-s period. Each reinforcer delivery was accompanied by a 1-s illumination of LED light within the nosepoke aperture and 1-s presentation of a tone. Following each infusion, a 20-s timeout period was enacted where additional active nosepokes were recorded but had no consequences. Nosepokes into the designated inactive aperture had no consequences at any time during the experiment. Prior to and following each 96-h session, catheters were flushed with 0.1 ml of heparinized Timentin as described above. Criteria for acquisition of α-PPP or MDPV self-administration were defined as the subject obtaining at least 50 infusions during the first 96-h session. The use of yoked administration of either drug or saline as a control was not employed, since yoked infusions have been shown to have aversive properties, as well as reduce the motivation for intake of psychostimulants (20, 21).

### Statistical Analyses

GraphPad Prism v.8.4 (GraphPad Software, La Jolla, CA) was utilized for statistical analyses. P-values <0.05 were considered statistically significant. A two-way mixed model analysis of variance (ANOVA) was used to analyze the number of α-PPP, MDPV or saline infusions obtained across each 96-h session, followed by Holm-Sidak corrections for multiple comparisons. We also analyzed the number of infusions obtained during this session in 8-h blocks to obtain a temporal profile of drug intake.

## Results

A total of n = 40 animals were implanted with intravenous catheters. Of these, data from n = 4 rats were excluded from analysis due to loss of catheter patency, and data from n = 5 rats were excluded from analysis due to failure to meet acquisition criteria (>100 infusions obtained during the first 96-h period, and/or failure to discriminate active vs. inactive nosepoke hole by a ratio of >1:1). Additionally, data from n = 2 rats were excluded from analyses due to the development of severe health problems, and one rat was found dead in the operant conditioning chamber during one of 96-h sessions. As a result, final group sizes were as follows: α-PPP 0.05 mg/kg/infusion (n = 8), α-PPP 0.1 mg/kg/infusion (n = 4), α-PPP 0.32 mg/kg/infusion (n = 4), MDPV (n = 6), and saline (n = 6).

**Figure 1:**
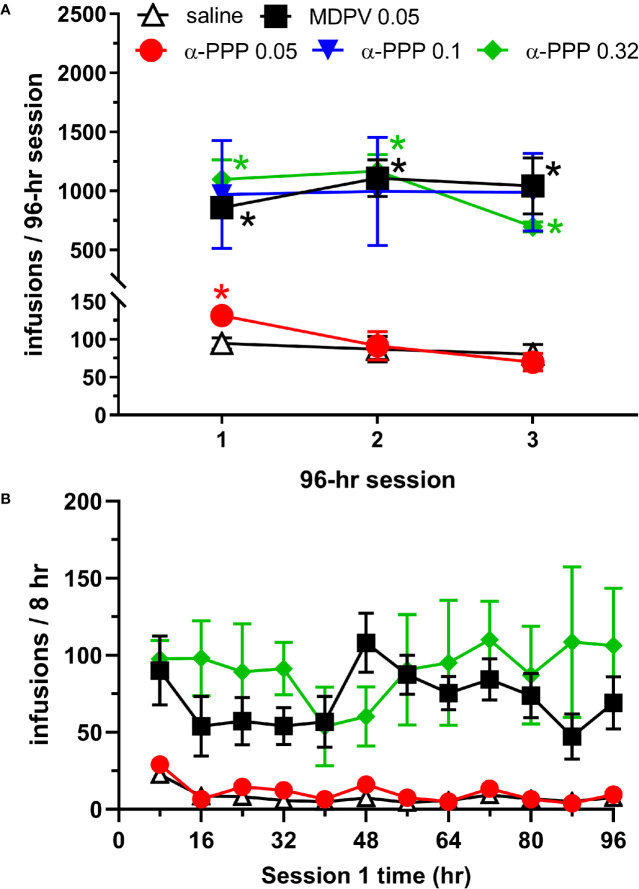
**(A)** Total infusions of saline (n = 6), MDPV (n = 6), or one of the three doses of α-PPP (0.05, 0.1. or 0.32 mg/kg/infusion, n = 8, 4, and 4, respectively) during each of the three 96-h self-administration sessions. * indicates p<0.05 vs. the number of saline infusions obtained during the same 96-h session. **(B)** Total number of infusions of α-PPP (0.05 and 0.32 mg/kg/infusion), MDPV, or saline obtained in 8-h time bins during the first 96-h self-administration session. See **Table 1** for statistical significance of individual groups and time points. Data from the 0.1 mg/kg/infusion group are not shown to enhance clarity of the data presented, and as a result of lack of statistically significant overall intake observed. All data are presented as mean ± SEM.

Analysis of the number of infusions earned across each of the three 96-h sessions revealed a significant effect of reinforcer (saline, MDPV or one of the 3 doses of α-PPP, F_4,23_ = 12.05, p<0.0001), session (F_1.61, 36.25_ = 4.29, p<0.05, and an interaction between these factors (F_8,45_ = 4.53, p<0.0005). Post hoc analyses revealed that the number of MDPV infusions obtained was greater than the number of saline infusions across all three 96-h sessions (p-values <0.0005, 0.005, and 0.05 during the first, second and third 96-h sessions, respectively). In animals self-administering the low (0.05 mg/kg/infusion) dose of α-PPP, the number of infusions obtained was greater than the number of saline infusions obtained only during the first 96-h session (p<0.05, **Figure 1A**). In animals self-administering the 0.1 mg/kg/infusion dose of α-PPP, increased variability of responding was observed, and intake of the drug was not statistically different from that of saline (p-values = 0.15, 0.26, and 0.13 during the 1^st^, 2^nd^, and 3^rd^ 96-h sessions, respectively). However, in animals self-administering the 0.32 mg/kg/infusion dose of α-PPP, the number of infusions obtained was greater than the number of saline infusions obtained during all three 96-h sessions (all p-values <0.05, **Figure 1A**).

To obtain a temporal profile of drug intake patterns, we analyzed the number of infusions obtained in 8-h bins during the first 96-h session (**Figure 1B**). We observed significant effects of reinforcer (F_4,23_ = 9.81, p<0.0001) and not session (p>0.05), but a significant interaction between these factors (F_44,253_ = 1.63, p<0.05). Multiple comparisons of the number of drug infusions obtained across each 8-h time bin are provided in **Table 1**. The number of MDPV reinforcers earned were significantly greater than those of saline during the 4^th^, 6^th^ through 10^th^, and 12^th^ time bins. The number of α-PPP (0.05 mg/kg/infusion) reinforcers earned were significantly greater than those of saline only during the 4^th^ time bin, and the number of α-PPP (0.32 mg/kg/infusion) reinforcers earned were significantly greater than those of saline only during the 1^st^ and 4^th^ time bins. Temporal patterns of self-administration of the 0.1 mg/kg/infusion dose of α-PPP was highly variable (not shown in **Figure 1B** for clarity of presentation of the other doses), and significant differences with respect to levels of saline self-administration were not observed (**Table 1**).

**Table 1:** Analysis of 8-h time bins for self-administration of MDPV or one of three doses of α-PPP vs. saline during the first 96-h session.

Time (h)	Comparison	P-value
0-8	saline vs. MDPV	0.0847
	saline vs. α-PPP 0.05 mg/kg/inf	0.1875
	saline vs. α-PPP 0.1 mg/kg/inf	0.1875
	saline vs. α-PPP 0.32 mg/kg/inf	0.0272*
9-16	saline vs. MDPV	0.1863
	saline vs. α-PPP 0.05 mg/kg/inf	0.5632
	saline vs. α-PPP 0.1 mg/kg/inf	0.2615
	saline vs. α-PPP 0.32 mg/kg/inf	0.1308
17-24	saline vs. MDPV	0.0955
	saline vs. α-PPP 0.05 mg/kg/inf	0.0958
	saline vs. α-PPP 0.1 mg/kg/inf	0.1587
	saline vs. α-PPP 0.32 mg/kg/inf	0.1540
25-32	saline vs. MDPV	0.0400*
	saline vs. α-PPP 0.05 mg/kg/inf	0.0400*
	saline vs. α-PPP 0.1 mg/kg/inf	0.3440
	saline vs. α-PPP 0.32 mg/kg/inf	0.0400*
33-40	saline vs. MDPV	0.0992
	saline vs. α-PPP 0.05 mg/kg/inf	0.3919
	saline vs. α-PPP 0.1 mg/kg/inf	0.3875
	saline vs. α-PPP 0.32 mg/kg/inf	0.3858
40-48	saline vs. MDPV	0.0128*
	saline vs. α-PPP 0.05 mg/kg/inf	0.0744
	saline vs. α-PPP 0.1 mg/kg/inf	0.1692
	saline vs. α-PPP 0.32 mg/kg/inf	0.1333
49-56	saline vs. MDPV	0.0052*
	saline vs. α-PPP 0.05 mg/kg/inf	0.2557
	saline vs. α-PPP 0.1 mg/kg/inf	0.2976
	saline vs. α-PPP 0.32 mg/kg/inf	0.2557
57-64	saline vs. MDPV	0.0052*
	saline vs. α-PPP 0.05 mg/kg/inf	0.7317
	saline vs. α-PPP 0.1 mg/kg/inf	0.3153
	saline vs. α-PPP 0.32 mg/kg/inf	0.3067
65-72	saline vs. MDPV	0.0096*
	saline vs. α-PPP 0.05 mg/kg/inf	0.0798
	saline vs. α-PPP 0.1 mg/kg/inf	0.3569
	saline vs. α-PPP 0.32 mg/kg/inf	0.0798
73-80	saline vs. MDPV	0.0210*
	saline vs. α-PPP 0.05 mg/kg/inf	0.9455
	saline vs. α-PPP 0.1 mg/kg/inf	0.5562
	saline vs. α-PPP 0.32 mg/kg/inf	0.2352
81-88	saline vs. MDPV	0.1349
	saline vs. α-PPP 0.05 mg/kg/inf	0.4373
	saline vs. α-PPP 0.1 mg/kg/inf	0.1349
	saline vs. α-PPP 0.32 mg/kg/inf	0.2346
89-96	saline vs. MDPV	0.0585
	saline vs. α-PPP 0.05 mg/kg/inf	0.5832
	saline vs. α-PPP 0.1 mg/kg/inf	0.5832
	saline vs. α-PPP 0.32 mg/kg/inf	0.2125

Asterisks indicate statistically significant differences vs. saline.

## Discussion

The current study confirms our previous findings that MDPV is robustly self-administered under conditions of prolonged access in 96-h sessions (18). However, self-administration of α-PPP above levels observed for saline were variable and dose-dependent, as reliable self-administration was only observed at the highest dose tested (0.32 mg/kg/infusion). These findings are consistent with those of other investigators showing the reinforcing effects of this synthetic cathinone under conditions of limited (90 min/day) access (15). Self-administration of α-PPP has also been reported to occur in humans (22), although we note that it is difficult to extrapolate dose-related phenomena across species. We did not observe significant self-administration of α-PPP at a dose of 0.1 mg/kg/infusion. However, the lack of statistical significance may have resulted from a low sample size for this group, as two of the four animals tested in this group showed significant levels of α-PPP self-administration (individual subject data not shown).

Both MDPV and α-PPP can elicit cocaine-like locomotor stimulant and discriminative stimulus effects (9, 11, 23), and exhibit cocaine-like inhibitory effects on presynaptic DAT and NET transporters (7, 24). However, the potency of α-PPP as a DAT inhibitor is approximately 70 times less than that of MDPV (25), which is likely reflected in the higher doses of α-PPP required to support self-administration, as observed in the present and previous studies (15). Interestingly, however, α-PPP does not show significant affinity for presynaptic serotonin transporters, but recent studies have revealed some MDMA-like discriminative stimulus effects of this cathinone derivative (12).

Additionally, it was recently reported that α-PPP exhibits antagonist and inverse agonist activity at type 2A serotonin (5-HT_2A_) receptors at physiologically relevant (i.e., nanomolar) concentrations (26). This mechanism of action may limit some of the psychoactive and/or reinforcing effects of α-PPP, as pharmacological antagonism of 5-HT_2A_ receptors attenuates some of the behavioral and neurochemical effects of cocaine (27–29). It is therefore possible that in addition to its lower affinity for DAT and/or NET as compared to MDPV, inhibitory actions at 5-HT_2A_ receptors may reduce the relative reinforcing effects of α-PPP. However, this notion needs to be empirically tested prior to drawing any firm conclusions.

Upon examination of α-PPP self-administration in 8-h time bins across the first 96-h session, we observed no significant differences in temporal intake of the higher dose (0.32 mg/kg/infusion) of this drug with respect to those of MDPV. Possibly as a result of a greater degree of variability in drug intake for this dose of α-PPP, the number of infusions obtained per 8-h bin were significantly elevated during the 1^st^ and 4^th^ time bins as compared to saline, as opposed to being significantly increased during the 4^th^, 6^th^ through 10^th^, and 12^th^ time bins for MDPV relative to saline. For the low dose of α-PPP tested (0.05 mg/kg/infusion), levels of intake were increased above those observed for saline only during the 4^th^ 8-h time bin. Interestingly, however, during this 4^th^ 8-h time bin, we observed robust self-administration of MDPV as well as two of the three doses of alpha-PPP tested (0.05 and 0.32 mg/kg/infusion). The reason why such effects were observed during this particular time period are currently unknown and require further investigation.

It is worth noting that both α-PPP and MDPV have metabolites with significant biological activity. For example, metabolites of α-PPP include 2′′-oxo-PPP, 4′-hydroxy-PPP, cathinone and norpseudoephedrine (30), some of which may accumulate and prolong the effects of the parent compound. Likewise, MDPV is metabolized to 3,4-dihydroxypyrovalerone and 4-hydroxy-3-methoxypyrovalerone among other biotransformation products, with the former metabolite showing significant (nanomolar) inhibitory activity at DAT and NET (6, 31). While specific drug metabolites were not measured in the present study during or after prolonged 96-h sessions, it is important to keep the potential contributions of such metabolites in mind when interpreting results from studies employing prolonged drug intake procedures.

In summary, the present study demonstrates that α-PPP possesses reinforcing effects in rats under conditions of extended drug access, albeit with lower potency and reinforcing efficacy as compared to those of MDPV. However, given that α-PPP is currently unscheduled by the U.S. Drug Enforcement Agency, and to our knowledge is not widely classified as a controlled substance in other countries worldwide, these findings suggest that this particular pyrovalerone cathinone derivative may have the potential for abuse.

## Data Availability Statement

The raw data supporting the conclusions of this article will be made available by the authors, without undue reservation.

## Ethics Statement

The animal study was reviewed and approved by Institutional Animal Care and Use Committee at Arizona State University.

## Author Contributions

EN and FO conceived and designed the experiments, and wrote the manuscript. EN and PO performed behavioral testing. FO performed the data analyses.

## Funding

This work was supported by Public Health Service grant DA042172 from the National Institute on Drug Abuse.

## Conflict of Interest

The authors declare that the research was conducted in the absence of any commercial or financial relationships that could be construed as a potential conflict of interest.

## References

[1] Simmons SJ Leyrer-Jackson JM Oliver CF Hicks C Muschamp JW Rawls SM DARK classics in chemical neuroscience: Cathinone-derived psychostimulants. ACS Chem Neurosci (2018) 9(10):2379–94. 10.1021/acschemneuro.8b00147 PMC6197900 29714473

[2] Karila L Megarbane B Cottencin O Lejoyeux M Synthetic cathinones: a new public health problem. Curr Neuropharmacol (2015) 13(1):12–20. 10.2174/1570159X13666141210224137 26074740 PMC4462036

[3] Zawilska JB Wojcieszak J a-Pyrrolidinophenones: a new wave of designer cathinones. Forensic Toxicol (2017) 35:201–16. 10.1007/s11419-016-0353-6

[4] Simmler LD Buser TA Donzelli M Schramm Y Dieu LH Huwyler J Pharmacological characterization of designer cathinones in vitro. Br J Pharmacol (2013) 168(2):458–70. 10.1111/j.1476-5381.2012.02145.x PMC3572571 22897747

[5] Rickli A Hoener MC Liechti ME Monoamine transporter and receptor interaction profiles of novel psychoactive substances: para-halogenated amphetamines and pyrovalerone cathinones. Eur Neuropsychopharmacol (2015) 25(3):365–76. 10.1016/j.euroneuro.2014.12.012 25624004

[6] Baumann MH Bukhari MO Lehner KR Anizan S Rice KC Concheiro M Neuropharmacology of 3,4-methylenedioxypyrovalerone (MDPV), its metabolites, and related analogs. Curr Top Behav Neurosci (2017) 32:93–117. 10.1007/7854_2016_53 27830575 PMC5392131

[7] Eshleman AJ Wolfrum KM Reed JF Kim SO Swanson T Johnson RA Structure-activity relationships of substituted cathinones, with transporter binding, uptake, and release. J Pharmacol Exp Ther (2017) 360(1):33–47. 10.1124/jpet.116.236349 27799294 PMC5193076

[8] Baumann MH Partilla JS Lehner KR Thorndike EB Hoffman AF Holy M Powerful cocaine-like actions of 3,4-methylenedioxypyrovalerone (MDPV), a principal constituent of psychoactive ‘bath salts’ products. Neuropsychopharmacology (2013) 38(4):552–62. 10.1038/npp.2012.204 PMC3572453 23072836

[9] Gatch MB Taylor CM Forster MJ Locomotor stimulant and discriminative stimulus effects of ‘bath salt’ cathinones. Behav Pharmacol (2013) 24(5-6):437–47. 10.1097/FBP.0b013e328364166d PMC4183201 23839026

[10] Gatch MB Rutledge MA Forster MJ Discriminative and locomotor effects of five synthetic cathinones in rats and mice. Psychopharmacology (2015) 232(7):1197–205. 10.1007/s00213-014-3755-3 PMC4361374 25281225

[11] Gatch MB Dolan SB Forster MJ Locomotor activity and discriminative stimulus effects of a novel series of synthetic cathinone analogs in mice and rats. Psychopharmacol (Berl) (2017) 234(8):1237–45. 10.1007/s00213-017-4562-4 PMC5364041 28210779

[12] Gatch MB Forster MJ Methylenedioxymethamphetamine-like discriminative stimulus effects of pyrrolidinyl cathinones in rats. J Psychopharmacol (2020) 34(7):778–85. 10.1177/0269881120914213 PMC11571078 32536334

[13] Watterson LR Kufahl PR Nemirovsky NE Sewalia K Grabenauer M Thomas BF Potent rewarding and reinforcing effects of the synthetic cathinone 3,4-methylenedioxypyrovalerone (MDPV). Addict Biol (2014) 19:165–74. 10.1111/j.1369-1600.2012.00474.x PMC3473160 22784198

[14] Aarde SM Huang PK Creehan KM Dickerson TJ Taffe MA The novel recreational drug 3,4-methylenedioxypyrovalerone (MDPV) is a potent psychomotor stimulant: self-administration and locomotor activity in rats. Neuropharmacology (2013) 71:130–40. 10.1016/j.neuropharm.2013.04.003 PMC3681807 23597511

[15] Gannon BM Galindo KI Mesmin MP Sulima A Rice KC Collins GT Relative reinforcing effects of second-generation synthetic cathinones: Acquisition of self-administration and fixed ratio dose-response curves in rats. Neuropharmacology (2018) 134(Pt A):28–35. 10.1016/j.neuropharm.2017.08.018 28811192 PMC5809320

[16] Miotto K Striebel J Cho AK Wang C Clinical and pharmacological aspects of bath salt use: a review of the literature and case reports. Drug Alcohol Depend (2013) 132(1-2):1–12. 10.1016/j.drugalcdep.2013.06.016 23916320

[17] Palamar JJ Martins SS Su MK Ompad DC Self-reported use of novel psychoactive substances in a US nationally representative survey: prevalence, correlates, and a call for new survey methods to prevent underreporting. Drug Alcohol Depend (2015) 156:112–9. 10.1016/j.drugalcdep.2015.08.028 PMC4633323 26377051

[18] Sewalia K Watterson LR Hryciw A Belloc A Ortiz JB Olive MF Neurocognitive dysfunction following repeated binge-like self-administration of the synthetic cathinone 3,4-methylenedioxypyrovalerone (MDPV). Neuropharmacology (2018) 134(Pt A):36–45. 10.1016/j.neuropharm.2017.11.034 29183686 PMC5966333

[19] Cornett EM Goeders NE 96-hour methamphetamine self-administration in male and female rats: A novel model of human methamphetamine addiction. Pharmacol Biochem Behav (2013) 111:51–7. 10.1016/j.pbb.2013.08.005 23958580

[20] Mutschler NH Miczek KA Withdrawal from a self-administered or non-contingent cocaine binge: differences in ultrasonic distress vocalizations in rats. Psychopharmacology (1998) 136(4):402–8. 10.1007/s002130050584 9600587

[21] Twining RC Bolan M Grigson PS Yoked delivery of cocaine is aversive and protects against the motivation for drug in rats. Behav Neurosci (2009) 123(4):913–25. 10.1037/a0016498 PMC3861880 19634952

[22] Sellors K Jones A Chan B Death due to intravenous use of α-pyrrolidinopentiophenone. Med J Aust (2014) 201(10):601–3. 10.5694/mja13.00203 25390268

[23] Ray A Chitre NM Daphney CM Blough BE Canal CE Murnane KS Effects of the second-generation “bath salt” cathinone alpha-pyrrolidinopropiophenone (α-PPP) on behavior and monoamine neurochemistry in male mice. Psychopharmacol (Berl) (2019) 236(3):1107–17. 10.1007/s00213-018-5044-z PMC6443494 30276421

[24] Marusich JA Antonazzo KR Wiley JL Blough BE Partilla JS Baumann MH Pharmacology of novel synthetic stimulants structurally related to the “bath salts” constituent 3,4-methylenedioxypyrovalerone (MDPV). Neuropharmacology (2014) 87:206–13. 10.1016/j.neuropharm.2014.02.016 PMC4152390 24594476

[25] Gannon BM Baumann MH Walther D Jimenez-Morigosa C Sulima A Rice KC The abuse-related effects of pyrrolidine-containing cathinones are related to their potency and selectivity to inhibit the dopamine transporter. Neuropsychopharmacology (2018) 43(12):2399–407. 10.1038/s41386-018-0209-3 PMC6180085 30305739

[26] Chen Y Blough BE Murnane KS Canal CE The synthetic cathinone psychostimulant α-PPP antagonizes serotonin 5-HT_2A_ receptors: In vitro and in vivo evidence. Drug Test Anal (2019) 11(7):990–8. 10.1002/dta.2582 PMC6615953 30845376

[27] O’Neill MF Heron-Maxwell CL Shaw G 5-HT_2_ receptor antagonism reduces hyperactivity induced by amphetamine, cocaine, and MK-801 but not D1 agonist c-APB. Pharmacol Biochem Behav (1999) 63(2):237–43. 10.1016/S0091-3057(98)00240-8 10371652

[28] McMahon LR Cunningham KA Antagonism of 5-hydroxytryptamine_2a_ receptors attenuates the behavioral effects of cocaine in rats. J Pharmacol Exp Ther (2001) 297(1):357–63. 11259563

[29] Murnane KS Winschel J Schmidt KT Stewart LM Rose SJ Cheng K Serotonin 2A receptors differentially contribute to abuse-related effects of cocaine and cocaine-induced nigrostriatal and mesolimbic dopamine overflow in nonhuman primates. J Neurosci (2013) 33(33):13367–74. 10.1523/jneurosci.1437-13.2013 PMC3742924 23946394

[30] Springer D Fritschi G Maurer HH Metabolism of the new designer drug alpha-pyrrolidinopropiophenone (PPP) and the toxicological detection of PPP and 4’-methyl-alpha-pyrrolidinopropiophenone (MPPP) studied in rat urine using gas chromatography-mass spectrometry. J Chromatogr B Analyt Technol BioMed Life Sci (2003) 796(2):253–66. 10.1016/j.jchromb.2003.07.008 14581066

[31] Meltzer PC Butler D Deschamps JR Madras BK 1-(4-methylphenyl)-2-pyrrolidin-1-yl-pentan-1-one (pyrovalerone) analogues: a promising class of monoamine uptake inhibitors. J Med Chem (2006) 49(4):1420–32. 10.1021/jm050797a PMC2602954 16480278

